# Poly(ADP-Ribose) Polymerase 1 (PARP1) Overexpression in Human Breast Cancer Stem Cells and Resistance to Olaparib

**DOI:** 10.1371/journal.pone.0104302

**Published:** 2014-08-21

**Authors:** Marine Gilabert, Simon Launay, Christophe Ginestier, François Bertucci, Stéphane Audebert, Mathieu Pophillat, Yves Toiron, Emilie Baudelet, Pascal Finetti, Tetsuro Noguchi, Hagay Sobol, Daniel Birnbaum, Jean-Paul Borg, Emmanuelle Charafe-Jauffret, Anthony Gonçalves

**Affiliations:** 1 Institut Paoli-Calmettes, Department of Molecular Pharmacology, Marseille, France; 2 Institut Paoli-Calmettes, Department of Medical Oncology, Marseille, France; 3 Institut Paoli-Calmettes, Department of Biopathology, Marseille, France; 4 Institut Paoli-Calmettes, Department of Molecular Oncology, Marseille, France; 5 Institut Paoli-Calmettes, Department of Cancer Genetics, Marseille, France; 6 Aix-Marseille University, Marseille, France; 7 Centre de Recherche en Cancérologie de Marseille, U1068 INSERM, U7258 CNRS, Marseille, France; Rutgers - New Jersey Medical School, United States of America

## Abstract

**Background:**

Breast cancer stem cells (BCSCs) have been recognized as playing a major role in various aspects of breast cancer biology. To identify specific biomarkers of BCSCs, we have performed comparative proteomics of BCSC-enriched and mature cancer cell populations from the human breast cancer cell line (BCL), BrCA-MZ-01.

**Methods:**

ALDEFLUOR assay was used to sort BCSC-enriched (ALDH+) and mature cancer (ALDH−) cell populations. Total proteins were extracted from both fractions and subjected to 2-Dimensional Difference In-Gel Electrophoresis (2-D DIGE). Differentially-expressed spots were excised and proteins were gel-extracted, digested and identified using MALDI-TOF MS.

**Results:**

2-D DIGE identified poly(ADP-ribose) polymerase 1 (PARP1) as overexpressed in ALDH+ cells from BrCA-MZ-01. This observation was confirmed by western blot and extended to four additional human BCLs. ALDH+ cells from BRCA1*-*mutated HCC1937, which had the highest level of PARP1 overexpression, displayed resistance to olaparib, a specific PARP1 inhibitor.

**Conclusion:**

An unbiased proteomic approach identified PARP1 as upregulated in ALDH+, BCSC-enriched cells from various human BCLs, which may contribute to clinical resistance to PARP inhibitors.

## Introduction

Breast cancer stem cells (BCSC), which are defined by their capacity of self-renewal and their ability to provide to non-tumorigenic, more differentiated cells, are increasingly recognized as major actors in breast cancer [1]. They are thought to be involved in critical aspects of breast cancer biology including carcinogenesis, metastasis, resistance to treatments, and thus clinical recurrence [2], [3]. Accordingly, an effective targeting of BCSC is expected to significantly improve breast cancer outcome. As we previously demonstrated, flow cytometry-based detection of aldehyde dehydrogenase (ALDH) activity, can sort a subpopulation of cells from patient-derived xenografts and human breast cancer cell lines (BCLs) that have BCSC properties [4], [5], including tumorsphere-forming capacity, serial passages in NOD/SCID mice and recapitulation of the cellular heterogeneity of the original cell lines.

Proteomics-based profiling technologies may provide an unbiased approach to identify differentially expressed proteins between BCSC and non-BCSC populations, which may allow a more accurate definition of the BCSC subpopulation, a better understanding of BCSC biology and eventually the identification of novel targets for BCSC-specific therapeutics. Using 2-dimensional Difference In-Gel Electrophoresis (2-D DIGE), we have compared BCSC-enriched ALDEFLUOR-positive (ALDH+) to ALDEFLUOR-negative (ALDH−) BrCA-MZ-01 human BCL and found a significant increased expression of Poly(ADP-ribose) polymerase 1 (PARP1), a promising target for a recently developed class of anticancer compounds.

## Materials and Methods

### Cell lines and culture conditions

BCLs were obtained from American Type Culture Collection (HCC1937, MDA-MB-436), from collections developed in the laboratory of Dr S. Ethier, Karmanos Cancer Institute, Detroit, Michigan, USA (SUM149 and SUM159; obtained from Asterand; UKhttp://www.asterand.com/Asterand/human_tissues/hubrcelllines.htm), and from Dr V.J. Möbus, University of Ulm, Ulm, Germany (BrCa-MZ-01) [6]. *BRCA1* and *BRCA2* gene status was checked for each cell line by full sequencing, as previously described [7]. The cell lines were grown using recommended culture conditions, as previously described [5].

### Aldehyde dehydrogenase activity detection and cell sorting

The ALDEFLUOR kit (StemCell Technologies) was used to isolate the population with high ALDH enzymatic activity using a FACStar PLUS (Becton Dickinson), as previously described [4], [5]. Briefly, cells were incubated in a specific buffer containing ALDH substrate, while a negative control sample was obtained by identical incubation in presence of a specific 50 mmol/L of diethylaminobenzaldehyde, a specific ALDH inhibitor. Cell viability was evaluated by propidium iodide and used to define the sorting gates.

### 2D-DIGE experiments

Proteins from ALDH+ and ALDH− BrCA-MZ-01 cell populations were extracted using an urea-based buffer (7.5 M urea, 0,01 ug/l DTT, 2.5 M thiourea, 12.,5 mM glycérol, 62,5 mM Tris-HCl 2,5%) containing 1,5 mM of a protease inhibitor cocktail (Sigma-Aldrich, USA) and an equal amount (50 µg) of each sample was labeled with either Cy3- (ALDH−), Cy5- (ALDH+) or Cy2- (internal standard) CyDye according to the manufacturer’s recommended protocols (GE Healthcare, Piscataway, NJ, USA), and pooled. Samples were then separated by 2-Dimensional gel electrophoresis with the following steps: isoelectric focusing, using 24-cm immobilized pH gradient strips (IPG 3–10 NL, GE Healthcare) and 2D-separation into 10% uniform polyacrylamyde gels. Gels with CyDye-labeled proteins were digitalized using the Typhoon Trio Image Scanner (GE Healthcare), images were cropped with ImageQuant software (GE Healthcare) and further analyzed using the DeCyder v 6.5 software package (GE healthcare). Spots of interest were excised, digested by trypsin and subjected to mass spectrometry analysis using MALDI-TOF MS (Ultraflex, Brucker Daltonics, Billerica, USA), using reflectron and positive modes with an ion acceleration of 25 keV. To process obtained mass spectra, we used the FlexAnalysis 2.0 software (Bruker Daltonics). To obtain protein identification, we used peptide mass fingerprint with an in-house Mascot server (Version 2.2.0, Matrix Science Inc., London, UK) probing the International Protein Index (IPI) human database from the European Bioinformatics Institute, as described in [8].

### Western blot experiments

Protein lysates were loaded into SDS-PAGE, transferred on nitrocellulose membrane, blocked 1 h at room temperature in Tris-Buffered Saline/5% non-fat dry milk/0,1% Tween20, and incubated overnight with primary antibodies in blocking solution (PARP1 and αTubulin mouse monoclonal antibodies, Sigma-Aldrich, USA). After extensive washings in TBS/0,1% Tween20, membranes were incubated 1 h at room temperature (RT) with a HRP-conjugated secondary antibody before being revealed with an enhanced chemiluminescence substrate (West Pico, Thermo Scientific, USA).

### Olaparib treatment

Twenty-four hours after seeding, medium was changed and MDA-MB-436, BrCA-MZ-01, SUM149, SUM159 and HCC1937 cells were grown during 72 hours either in the presence of an inhibitor of PARP, Olaparib (AZD2281, Euromedex, France) at 10 µM (according to their respective IC50%) or in the corresponding concentrations of DMSO (control). ALDH+ and ALDH− cells from treated and control cell lines were sorted as described above and absolute cell numbers were counted using trypan blue.

## Results

**Figure 1:**
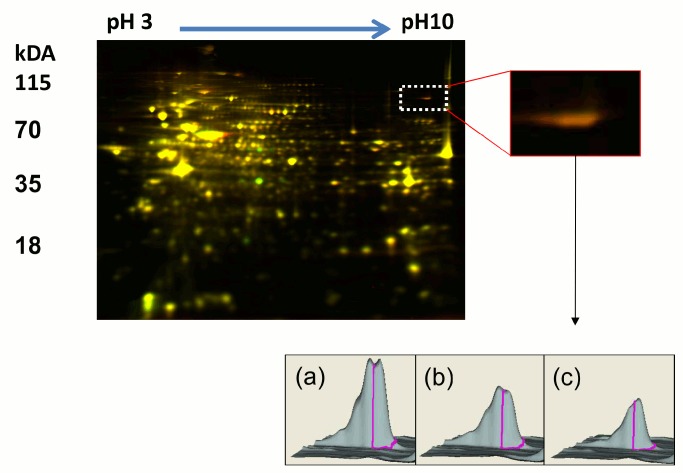
2D-DIGE analysis of ALDH+ versus ALDH− BrCA-MZ-01 cells. Upper panel, Representatives three-(left) and two-(right) color merged images of 2D-DIGE gel: red spots are from the Cy5-labeled ALDH+ cells, green spots are from Cy3-labeled ALDH− cells and blue spots are from standardized samples. **Lower panel,** Three-dimensional simulation of the relative abundance of PARP1 protein in ALDH+ (a), standardized (b) and ALDH− samples (c).

We used ALDEFLUOR-assay to sort ALDH+ and ALDH− cells from the *BRCA1*-mutated BrCA-MZ-01 human BCL and compared protein lysates obtained from these subpopulations by 2D-DIGE (Figure 1). Among the proteins with differential expression (Table S1), PARP1 was found as the most up-regulated one in ALDH+ cells (ratio ALDH+/ALDH− = 1.56, 20 peptides matched, 23% of sequence coverage).

**Figure 2:**
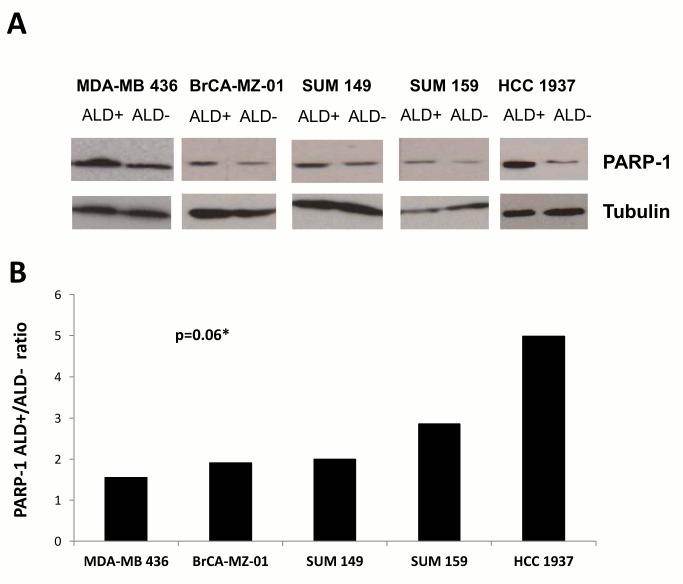
Up-regulated expression of PARP1 in ALDH+ relative to ALDH− cells from human breast cancer cell lines. **A**. Western blotting image. Presented blots are representative of at least 2 independent experiments. **B**. The quantitative comparison of PARP1 expression between ALDH+ and ALDH− cells, expressed as ratio. PARP1 protein expression was first normalized to Tubulin expression. * PARP1 protein expression was compared between ALDH+ and ALDH− cells using Wilcoxon signed rank test.

PARP1 protein expression was comparatively examined by western blot analysis in ALDH+ and ALDH− cell subpopulations from four additional human BCLs, including 3 other *BRCA1*-mutated BCLs (HCC1937, MDA-MB-436 and SUM149) and the *BRCA1/2*-wildtype SUM159 BCL (Figure 2). Overexpression of PARP1 in BCSC-enriched ALDH+ population was confirmed in all samples, with a ratio ALDH+/ALDH− ranging from 1.59 in MDA-MB-436 to 4.99 in HCC1937 cells. In spite of a limited number of samples, this increase approached statistical significance (p = 0.06, Wilcoxon signed rank test).

**Figure 3:**
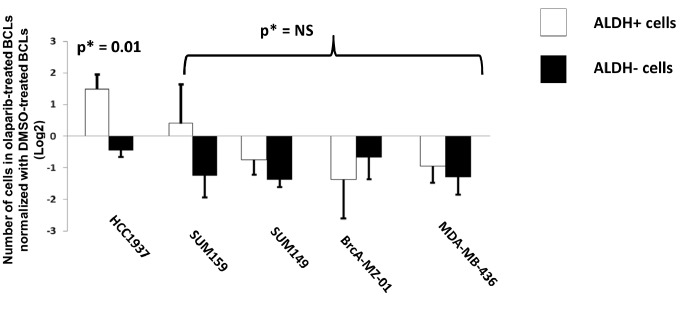
Effect of olaparib treatment on BCSCs from BCLs according to PARP1 expression. Human BCLs were seeded in culture flask and incubated 72 hours in olaparib- or DMSO-containing medium. At each time and for each condition, percentage of ALDH+ and ALDH− cells were determined using ALDEFLUOR assay and absolute number of cells was counted using trypan blue. Results are presented after normalization by numbers of cells in DMSO-treated conditions and Log-2 transformation. * Mean values of ALDH+ and ALDH− cells were compared using T-test.

To determine the potential impact of PARP1 overexpression on the sensitivity of BCSC-enriched cell subpopulation to PARP inhibitors, we exposed BCLs to 10 µm olaparib during 72 hours and monitored the absolute number of ALDH+ and ALDH− cells. As shown in Figure 3, whereas olaparib activity was not significantly different between ALDH+ and ALDH− cells from MDA-MB-436, BrCA-MZ-01, SUM149 and SUM 159, the absolute number of ALDH+ cells in HCC1937, which displayed the highest level of PARP1 overexpression, significantly increased, suggesting resistance to olaparib. In fact, there was a positive correlation between PARP1 overexpression in ALDH+ cells and their relative resistance to olaparib (Figure S1).

## Discussion

One of the main implication of BCSCs in breast cancer biology may be their potential role in therapeutic resistance [9]. Indeed, resistance to treatment, including chemotherapy and/or radiotherapy, was largely described in BCSCs and was thought to be related to various causes such as increased expression of adenosine triphosphate-binding cassette (ABC) transporters, resistance to apoptosis, lower proliferation and improved DNA repair ability [3]. By an unbiased proteomic-based approach, we have found an overexpression of PARP1 in BCSC-enriched ALDH+ cell subpopulations from various BCLs. PARP1, the most abundant isoform of the PARP superfamily, is a chromatin-associated protein which participates in various biological functions such as cell proliferation, apoptosis, malignant transformation, transcriptional regulation and DNA repair [10]. Thus, PARP1 plays a major role in base excision repair of DNA single-strand breaks. When DNA is damaged, PARP1 recognizes the lesion and is able to bind it. Then, PARP1 is activated and catalyzes poly(ADP-ribosylation) of various nuclear proteins. Ultimately, proteins modified by PARP1 activity will aggregate and activate major components of DNA repair pathways. Notably, recent data, including our own [11], have demonstrated an adverse prognostic impact of PARP1 overexpression in breast cancer [12].

In the absence of PARP1, DNA single-strand breaks accumulate and lead to DNA double-strand breaks, which are not appropriately repaired if the BRCA pathway is deficient or dysfunctional, a phenomenon called synthetic lethality [13], [14]. Consequently, PARP inhibition was recently studied in various cancers, including breast cancer, with contrasted results. In *BRCA1*- or *BRCA2*-mutated breast cancer, significant but transient and inconstant objective responses have been observed to olaparib in some studies [15], [16], while other results were less conclusive [17]. Moreover, after promising results in a randomized phase II trial [17], iniparib (the actual anti-PARP activity of which remains discussed) in combination with chemotherapy failed to demonstrate any survival advantage in triple-negative metastatic breast cancer [18], a subset of tumors sharing some similarities with *BRCA*-mutated breast cancer. In the present study, we have observed olaparib resistance of ALDH+ cells from the *BRCA1*-mutated HCC1937 BCL, which was associated with the highest level of PARP1 overexpression. A potential explanation to this observation could be that PARP1 overexpression improves DNA-repair capacity of BCSC, and thus favors resistance to DNA-damaging treatments, including olaparib. A similar phenomenon was suggested by a previous study in glioma cells showing that CD133+ cells, which are supposed to be enriched in stem cells, expressed higher levels of the DNA repair protein O6-methylguanine-DNA methyltransferase (MGMT) than CD133-, which correlated with resistance to radiation [19]. It can be argued that overexpression of a drug target usually results in drug sensitivity rather than resistance. However, if this holds true with some targeted therapeutics such as trastuzumab or endocrine therapy, there are also other examples of drug resistance associated with overexpression of the target, such as BCR-ABL with imatinib in chronic myeloid leukemias or DHFR with methotrexate in sarcomas [20], [21]. In these latter examples, and possibly with PARP1, overexpression of the target could increase the drug concentration required to effectively inhibit it. Another possible cause for increased resistance in ALDH+ cells could be overexpression of ABC transporters [22], which may function as drug-efflux proteins for various substrates, including olaparib [23]. However, by comparing gene expression profiles previously obtained from ALDH+ and ALDH− populations of the studied cell lines [5], we did not identify any significant difference between expression levels of ABC gene family (Figure S2). Identical results were found when restricting the comparison to the ALDH+ population of single HCC1937 cell line versus other cell lines, although such an analysis has an obvious limited statistical power (data not shown).

An important limitation of this study relies upon the small number of cell lines examined. Actually, only in HCC1937 cells, which display the highest differential expression of PARP1 between ALDH+ and ALDH− subsets, was observed a significant increase in BCSC population under olaparib. Thus, PARP1 overexpression may impede drug effect only above a certain threshold and this mechanism of resistance may only apply to some but not all breast cancers. This is consistent with clinical data showing exquisite antitumor activity for PARP inhibitors only in a subset of patients, even with BRCA-mutated tumors [15]–[17].

Clearly, the clinical relevance of our observations remains to be established and it is not known whether such a potential mechanism is actually operating in some BRCA-mutated breast cancer patients treated with olaparib. However, our results suggest that some intrinsic properties of BCSC such as increased DNA-repair capacity, may contribute to resistance to PARP inhibitors, providing a basis for the relative failure of their clinical development in breast cancer.

## Supporting Information

Figure S1 **Effect of olaparib treatment on BCSCs from BCLs according to PARP1 expression.** ALDH+/ALDH− ratios of PARP1 protein expression were plotted against the number of ALDH+ cells after olaparib treatment (normalized by DMSO-treated cells) in various BCLs. * Pearson correlation coefficient.
(TIF)

Figure S2 **mRNA expression of ABC family members in ALDH+ versus ALDH− cells.** Gene expression data were obtained using Affymetrix U133 Plus 2.0 human oligonucleotide microarrays as described in [5]. Left panel, Heat map showing gene expression of ABC family members in ALDH+ (black box) versus ALDH− cells (white box). * indicates ABC genes previously described as involved in drug-efflux [22]. Right panel, vulcano plot showing log2 ratio of fold-change in gene expression of ALDH+ versus ALDH− (x axis) versus - log 10 ratio of p-values of Mann-Whitney U (y axis).
(TIF)

Table S1 **Differentially expressed proteins in ALDH+ compared to ALDH− BrCA-MZ-01 cells, identified by 2D-DIGE and MS.**
(DOCX)

## References

[1] Wicha MS, Liu S, Dontu G (2006) Cancer stem cells: an old idea–a paradigm shift. Cancer Res 66: 1883–1890. 16488983 10.1158/0008-5472.CAN-05-3153

[2] Kakarala M, Wicha MS (2008) Implications of the cancer stem-cell hypothesis for breast cancer prevention and therapy. J Clin Oncol 26: 2813–2820 10.1200/JCO.2008.16.3931 18539959 PMC2789399

[3] Dean M, Fojo T, Bates S (2005) Tumour stem cells and drug resistance. Nat Rev Cancer 5: 275–284 10.1038/nrc1590 15803154

[4] Ginestier C, Hur MH, Charafe-Jauffret E, Monville F, Dutcher J, et al (2007) ALDH1 Is a Marker of Normal and Malignant Human Mammary Stem Cells and a Predictor of Poor Clinical Outcome. Cell Stem Cell 1: 555–567. 18371393 10.1016/j.stem.2007.08.014 PMC2423808

[5] Charafe-Jauffret E, Ginestier C, Iovino F, Wicinski J, Cervera N, et al (2009) Breast cancer cell lines contain functional cancer stem cells with metastatic capacity and a distinct molecular signature. Cancer Res 69: 1302–1313. 19190339 10.1158/0008-5472.CAN-08-2741 PMC2819227

[6] Möbus VJ, Moll R, Gerharz CD, Kieback DG, Merk O, et al (1998) Differential characteristics of two new tumorigenic cell lines of human breast carcinoma origin. Int J Cancer 77: 415–423. 9663605 10.1002/(sici)1097-0215(19980729)77:3<415::aid-ijc18>3.0.co;2-6

[7] Hassanein M, Huiart L, Bourdon V, Rabayrol L, Geneix J, et al (2013) Prediction of BRCA1 germ-line mutation status in patients with breast cancer using histoprognosis grade, MS110, Lys27H3, vimentin, and KI67. Pathobiology 80: 219–227 10.1159/000339432 23614934

[8] Goncalves A, Charafe-Jauffret E, Bertucci F, Audebert S, Toiron Y, et al (2008) Protein profiling of human breast tumor cells identifies novel biomarkers associated with molecular subtypes. Mol Cell Proteomics 7: 1420–1433. 18426791 10.1074/mcp.M700487-MCP200 PMC2500227

[9] Eyler CE, Rich JN (2008) Survival of the Fittest: Cancer Stem Cells in Therapeutic Resistance and Angiogenesis. J Clin Oncol 26: 2839–2845 10.1200/JCO.2007.15.1829 18539962 PMC2739000

[10] Ame JC, Spenlehauer C, de Murcia G (2004) The PARP superfamily. Bioessays 26: 882–893. 15273990 10.1002/bies.20085

[11] Gonçalves A, Finetti P, Sabatier R, Gilabert M, Adelaide J, et al (2011) Poly(ADP-ribose) polymerase-1 mRNA expression in human breast cancer: a meta-analysis. Breast Cancer Res Treat 127: 273–281 10.1007/s10549-010-1199-y 21069454

[12] Von Minckwitz G, Müller BM, Loibl S, Budczies J, Hanusch C, et al (2011) Cytoplasmic poly(adenosine diphosphate-ribose) polymerase expression is predictive and prognostic in patients with breast cancer treated with neoadjuvant chemotherapy. J Clin Oncol 29: 2150–2157 10.1200/JCO.2010.31.9079 21519019

[13] Bryant HE, Schultz N, Thomas HD, Parker KM, Flower D, et al (2005) Specific killing of BRCA2-deficient tumours with inhibitors of poly(ADP-ribose) polymerase. Nature 434: 913–917. 15829966 10.1038/nature03443

[14] Farmer H, McCabe N, Lord CJ, Tutt ANJ, Johnson DA, et al (2005) Targeting the DNA repair defect in BRCA mutant cells as a therapeutic strategy. Nature 434: 917–921. 15829967 10.1038/nature03445

[15] Fong PC, Boss DS, Yap TA, Tutt A, Wu P, et al (2009) Inhibition of poly(ADP-ribose) polymerase in tumors from BRCA mutation carriers. N Engl J Med 361: 123–134 10.1056/NEJMoa0900212 19553641

[16] Tutt A, Robson M, Garber JE, Domchek SM, Audeh MW, et al (2010) Oral poly(ADP-ribose) polymerase inhibitor olaparib in patients with BRCA1 or BRCA2 mutations and advanced breast cancer: a proof-of-concept trial. The Lancet 376: 235–244. 10.1016/S0140-6736(10)60892-6 20609467

[17] Gelmon KA, Tischkowitz M, Mackay H, Swenerton K, Robidoux A, et al (2011) Olaparib in patients with recurrent high-grade serous or poorly differentiated ovarian carcinoma or triple-negative breast cancer: a phase 2, multicentre, open-label, non-randomised study. Lancet Oncol 12: 852–861 10.1016/S1470-2045(11)70214-5 21862407

[18] O’Shaughnessy J, Schwartzberg L, Danso MR, Miller K, Yardley D, et al.. (2011) A randomized phase III study of iniparib (BSI-201) in combination with gemcitabine/carboplatin (G/C) in metastatic triple-negative breast cancer (TNBC). J Clin Oncol 29 suppl; abstr 1007.

[19] Bao S, Wu Q, McLendon RE, Hao Y, Shi Q, et al (2006) Glioma stem cells promote radioresistance by preferential activation of the DNA damage response. Nature 444: 756–760 10.1038/nature05236 17051156

[20] Barnes DJ, Palaiologou D, Panousopoulou E, Schultheis B, Yong ASM, et al (2005) Bcr-Abl expression levels determine the rate of development of resistance to imatinib mesylate in chronic myeloid leukemia. Cancer Res 65: 8912–8919 10.1158/0008-5472.CAN-05-0076 16204063

[21] Guo W, Healey JH, Meyers PA, Ladanyi M, Huvos AG, et al (1999) Mechanisms of Methotrexate Resistance in Osteosarcoma. Clinical Cancer Research 5: 621–627. 10100715

[22] Dean M (2009) ABC transporters, drug resistance, and cancer stem cells. J Mammary Gland Biol Neoplasia 14: 3–9 10.1007/s10911-009-9109-9 19224345

[23] Kelly RJ, Robey RW, Chen CC, Draper D, Luchenko V, et al (2012) A pharmacodynamic study of the P-glycoprotein antagonist CBT-1 in combination with paclitaxel in solid tumors. Oncologist 17: 512 10.1634/theoncologist.2012-0080 22416063 PMC3336838

