# Supplementary figures and images for: Poly(ADP-Ribose) Polymerase 1 (PARP1) Overexpression in Human Breast Cancer Stem Cells and Resistance to Olaparib
Source: PLoS One. 2014 Aug 21;9(8):e104302. doi: 10.1371/journal.pone.0104302 (PMC4140711; doi:10.1371/journal.pone.0104302)

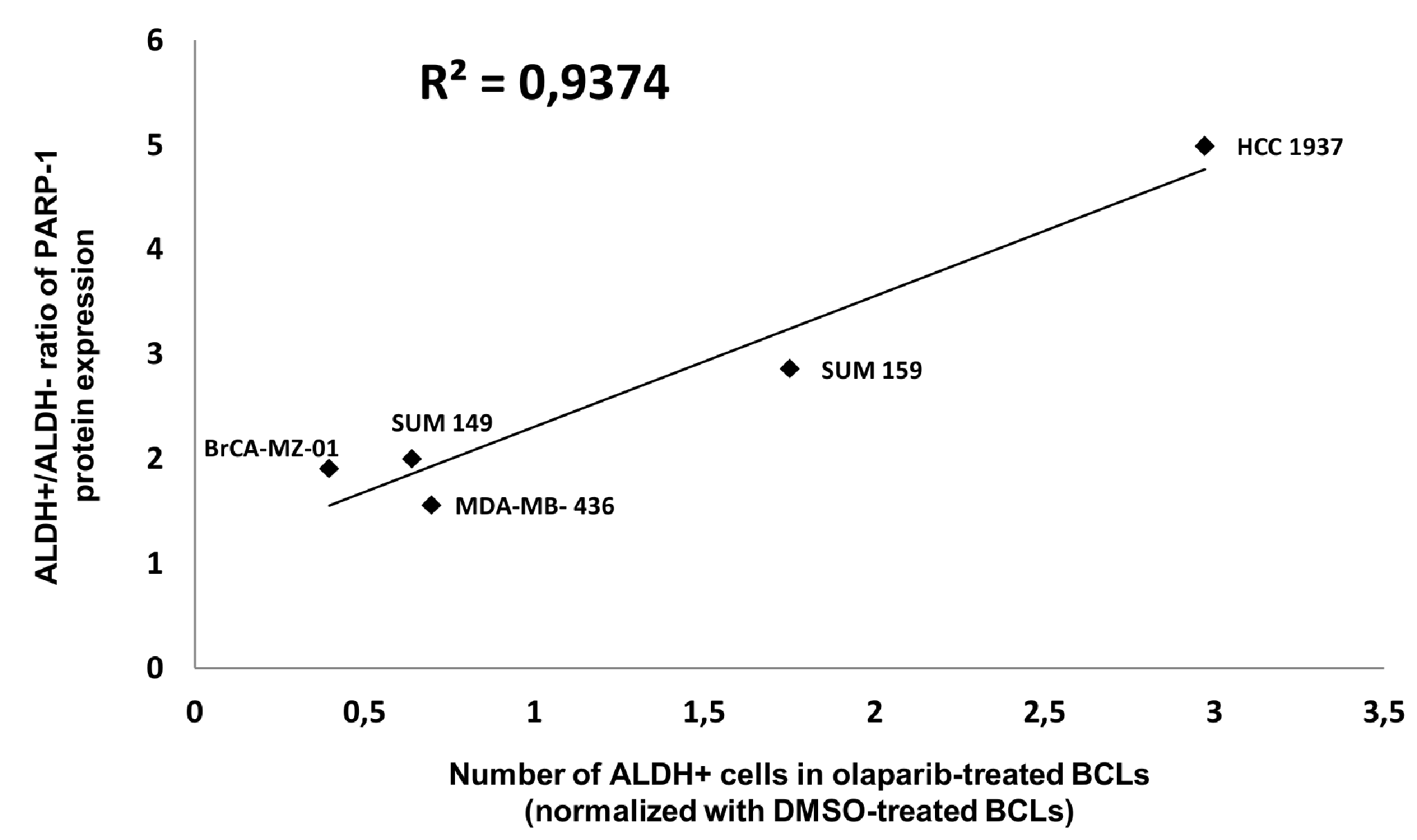

Supplement: Figure S1 — Effect of olaparib treatment on BCSCs from BCLs according to PARP1 expression. ALDH+/ALDH− ratios of PARP1 protein expression were plotted against the number of ALDH+ cells after olaparib treatment (normalized by DMSO-treated cells) in various BCLs. * Pearson correlation coefficient. (TIF) [file pone.0104302.s001.tif]
