# Supplementary material for: Poly(ADP-Ribose) Polymerase 1 (PARP1) Overexpression in Human Breast Cancer Stem Cells and Resistance to Olaparib
Source: PLoS One. 2014 Aug 21;9(8):e104302. doi: 10.1371/journal.pone.0104302 (PMC4140711; doi:10.1371/journal.pone.0104302)
Supplement: Table S1 — Differentially expressed proteins in ALDH+ compared to ALDH− BrCA-MZ-01 cells, identified by 2D-DIGE and MS. (DOCX) [file pone.0104302.s003.docx]

| **Proteins** | **Mass (Da)** | **Database** | **MOI** | **NMP** | **Sequence coverage (%)** | **Average ratio** |
| --- | --- | --- | --- | --- | --- | --- |
| Fatty acid synthase (FAS) | 275850 | SWISSPROT | PMF | 52 | 28 | + 1.51 |
| Leucine-rich PPR motif-containing domain (LPPRC) | 159003 | SWISSPROT | PMF | 21 | 19 | +1.55 |
| Phosphoribosyl formyl glycinamidine synthase (PUR4) | 146286 | SWISSPROT | PMF | 11 | 12 | + 1.55 |
| Poly(ADP-ribose) polymerase 1 (PARP-1) | 113811 | SWISSPROT | PMF | 20 | 23 | + 1.56 |
| Annexin A11 (ANXA11) | 54697 | SWISSPROT | PMF | 10 | 23 | - 3.29 |
| Heterogeneous nuclear ribonucleoprotein H (HNRH1) | 49484 | SWISSPROT | PMF | 9 | 35 | - 2.19 |
| Cytosol Aminopeptidase (AMPL) | 56530 | SWISSPROT | PMF | 10 | 21 | - 2.19 |
| Retinal dehydrogenase 1 (ALIA1) | 55454 | SWISSPROT | PMF | 9 | 21 | - 2.19 |
| Alpha-enolase (ENOA) | 47481 | SWISSPROT | PMF | 9 | 21 | - 1.53 |
| Annexin A1 (ANXA1) | 38918 | SWISSPROT | PMF | 8 | 30 | - 3.26 |
| Annexin A2 (ANXA2) | 38918 | SWISSPROT | PMF | 9 | 34 | - 2.35 |
| Annexin A3 (ANXA3) | 38918 | SWISSPROT | PMF | 12 | 48 | - 3.26 |
| Cofilin-1 (COF1) | 18719 | SWISSPROT | PMF | 4 | 29 | - 3.15 |

**Supplementary Table 1. Differentially expressed proteins in ALDH+ compared to ALDH- BrCA-MZ-01 cells, identified by 2D-DIGE and MS**

NMP, number of matched peptides. MOI, methods of identification. PMF, peptide mass fingerprint; Ratio = ALDH+/ALDH-; +, up-regulated in ALDH+ cells; -, down-regulated in ALDH+ cells)
